# The Missing Pieces of the Puzzle: A Review on the Interactive Nature of A-Priori Expectancies and Attention Bias toward Threat

**DOI:** 10.3390/brainsci10100745

**Published:** 2020-10-17

**Authors:** Elinor Abado, Tatjana Aue, Hadas Okon-Singer

**Affiliations:** 1 School of Psychological Sciences, University of Haifa, 3498838 Haifa, Israel; hadasos@psy.haifa.ac.il; 2 The Integrated Brain and Behavior Research Center (IBBR), University of Haifa, 3498838 Haifa, Israel; 3 Department of Psychology, University of Bern, 3012 Bern, Switzerland; tatjana.aue@psy.unibe.ch

**Keywords:** attention bias, expectancy attention bias modification, anxiety disorders, emotion

## Abstract

The role of attention bias in the etiology and maintenance of anxiety disorders has been studied extensively over decades. Attention bias reflects maladaptation in cognitive processing, as perceived threatening stimuli receive prioritized processing even when they are task-irrelevant or factually unthreatening. Recently, there has been some interest in the role of a-priori expectancies in attention bias toward threat. The current review article will present recent studies as examples that emphasize the need for more comprehensive research about the interactive effects of various factors that affect the relationship between expectancies and attention bias toward threatening stimuli in anxiety. The current review article suggests a holistic view, which advocates for more integrative research, as a dynamic network could underlie changes in attention bias. The study of the interaction between such factors, with a focus on expectancy, can lead to more ecological and clinically important results, and thus to more informed and fine-tuned treatments that are based on manipulation of expectancies. Such methods, in turn, can also help in shedding light on the research of attention bias, in a mutual relationship between research and therapy.

## 1. Attention Bias toward Threat: Manifestations and Interactions with Other Cognitive Biases

Attention bias toward threatening stimuli is defined as the disproportionate allocation of attentional resources toward potentially threatening stimuli (for a review, see [1,2]). For instance, a person with snake phobia might fixate on a picture of a snake, or on a previous location of this picture on a computer screen. This reaction could be considered unnecessary and excessive, especially in the experimental setting, where participants are asked to respond as quickly as possible to a neutral task-relevant target and ignore task-irrelevant threatening information. In this case, staring at a picture is a waste of attentional resources and leads to a decline in performance, as one fails to keep up with experimental demands. However, if one sees a snake while taking a hike, taking note of its features (e.g., size, colors, pattern and speed) may be of value. Attention bias is believed to play a major role in the etiology and in the maintenance of anxiety disorders [3]. Therefore, studying the relation between attention bias and anxiety is of high importance, from theoretical and clinical points of view.

Attention bias is suggested to consist of three components: faster engagement with threatening stimuli compared to neutral stimuli; slower disengagement from threatening stimuli compared to neutral stimuli; finally, following these initial components of vigilance, there is an avoidance component, in which participants avoid the fearful stimulus [4]. Several paradigms are used to study different components of attention bias, and they usually involve the detection of some kind of stimulus (for a review, see [1]). For instance, visual search paradigms measure engagement, as participants may view a visual search array containing several distractors (e.g., neutral) and one deviant (e.g., threatening stimulus). The task is to detect the deviant picture as quickly and as accurately as possible. Results usually show that detection is faster (i.e., engagement is faster) when a threatening picture is embedded between neutral distractors, compared to when a neutral picture is embedded in neutral distractors. Albeit plenty of research has focused on attention bias, it is usually studied in isolation. This sort of approach makes sense both theoretically and methodologically, as research often aims to isolate variables in order to better understand their specific function without any intervening factors. Nonetheless, more data regarding factors that affect attention bias are needed in order to identify causality in the dynamic interplay between different elements. Importantly, such identification of causality may uncover starting points for clinical intervention. Specifically, research on the interaction between difference factors that modulate attention bias is scarce.

Recently, some studies have been focusing on the interactions between biases. For instance, Klein et al. [5] found that attention bias, interpretation bias, and fear-related associations each predict unique variance in avoidance of spiders in spider fearful children. In addition, a relation was found between size and distance estimations and fear of spiders, showing that fear may further be characterized by a perceptual bias [5]. Another approach aims at integrating attention, perception and motor behavior in anxiety, and combining individual characteristics with environmental ones (for a review, see [6]). Parsons, Booth, Songco, and Fox [7] further used a moderated network modelling approach in order to model the complex interactions between positive and negative memory and interpretation biases and their relation to mental health. Attention bias was meant to be included as well but was omitted from analysis due to reliability issues. Results showed a negative relationship between network connectivity and positive mental health [7]. Together, these results show that different cognitive biases can complement each other. Thus, there is an added benefit to the study of more than one bias, with or without an overlap.

In depression, Everaert, Duyck, and Koster [8] found inter-relations between attention, interpretation, and memory biases, such that memory depends on attention and interpretation of stimuli (for more details regarding the combined cognitive biases hypothesis, see [9]; for an extension of this hypothesis, see [10]). Hence, while Klein et al. [5] found little overlap between the biases in fear of spiders, Everaert et al. [8] found significant interactions between the same biases in depression. These studies suggest a dissociation between anxiety and depression in terms of interactions (or lack thereof) between cognitive biases (see also [11] for more on the overlap between cognitive biases in depression; see also [12] for a recent study on various cognitive biases in depression and anxiety).

The study of the interaction between cognitive biases is of great theoretical and clinical importance, and thus it has been receiving more and more attention in recent years. The interaction between cognitive, affective, motivational, and individual factors that affect attention bias can also benefit from such examinations. The aim of the present review article is to present an overview of relevant recent studies and to propose that the study of attention bias should not only examine factors that might modulate attention individually, but rather interactively, with a focus on expectancy and expectancy bias.

Several studies have examined the effects of a-priori expectancies on attention and attention bias. A-priori expectancies reflect individuals’ pre-conceived notions regarding certain stimuli and situations. Specifically, there are two types of biased expectancies: encounter bias, which reflects the tendency of participants with anxiety to over-estimate the likelihood of encountering threat-relevant stimuli; and consequence bias, which reflect the tendency of individuals with anxiety to overestimate the negative outcomes which would accompany the encounter with threat-relevant stimuli. Furthermore, there are biased a-posteriori expectancies, which are exhibited after the exposure to threat-relevant stimuli. These are also known as memory biases (for a review, see [10]; for a review on expectancies toward positive stimuli, see [13]; for a review on expectancies toward negative stimuli, see [14]). Expectancies can be measured and manipulated in various ways, implicitly and explicitly. Additionally, biased expectancies to encounter threatening stimuli can be a trait characteristic, as will be discussed below.

In the current review article, therefore, we present several cognitive, affective, and personality factors, which affect the relation between expectancy and attention. We discuss recent findings in order to clearly illustrate the complexity and the dynamic network which might modulate attentional biases and their relation to expectancy. The current review article further aims at offering several suggestions for new directions in the field of attention bias and attention bias modification (ABM), and specifically on ways in which expectancies can affect and reduce attention bias. The discussed factors may act in unison or they may complement each other, depending on the disorder. Thus, mapping the varying factors and the different interactions between them across disorders may help clinicians to accurately diagnose and to effectively treat these disorders. This can be especially beneficial for the development of more effective ABM treatments that aim to reduce anxiety symptoms by reducing attention bias. In subsequent sections, we will present several examples of critical factors that could modulate attention bias using a better understanding of a-priori expectancies. Some of the presented studies do not relate to attention bias per se, but instead refer to prioritized processing of emotional stimuli, which is key in attention bias and in cognitive biases in general (for review on the dynamic modulation of emotional processing, see [15]).

## 2. The Influence of Exogenous vs. Endogenous Factors on Attention Bias to Threat

A recent opinion article emphasized the importance of integrating “goal-directed cognitive control” and “salience-driven” processes when trying to reduce attention bias using ABM [16]. Goal-directed cognitive control processes refer to “multiple processes supporting goal-directed thought and action. It includes attention control functions (goal-directed inhibitory attention control, flexible switching, controlled orienting), as well as reason-based evaluation/appraisal, and updating and maintaining information in working memory” ([16]; p. 226). These processes can also be referred to as “top-down” executive control processes. Salience-driven processes, on the other hand, refer to the fact that “stimulus salience varies along various dimensions including aversive (threat-related) and appetitive (reward-related) motivational salience, and perceptual salience (e.g., brightness, contrast). Motivational salience is influenced by stimulus content, individual differences, and situational variables (e.g., learning history, current motivational state, context)” ([16], p. 227). These processes can also be referred to as “bottom-up” processes. Both types of processes are interactive and complex, and include many different variables that affect attention bias. Notably, Mogg & Bradley [16] included learning history as a salience-driven process. Awh, Belopolsky and Theeuwes [17] suggested that a dichotomy between top-down and bottom-up factors is too simplistic, as some factors, such as selection history, are both top-down and bottom-up, depending on current goals and physical salience. Here, we will use the terms “endogenous” and “exogenous” factors, reflecting processes that are more internally generated or more externally generated, respectively.

In their review on cognitive mechanisms underlying anxiety and its treatment with ABM, Mogg, and Bradley [18] suggested an integrative framework. According to this framework, there is an imbalance between cognitive control and stimulus-related factors in anxiety, thereby launching a “threat-focused motivation mode” (p. 89). This mode has been proposed to include non-adaptive patterns of processing and reactions in various domains, such as attention, memory, reasoning, and stimulus-evaluation. In the study of attention bias, many studies focus on exogenous factors, such as pop-out and salience, which capture attention. For instance, approximately two decades ago, Öhman, Flykt, and Esteves [18] pointed out that snakes can be salient and pop-out, especially among snake fearful participants. More recent studies have also found that participants can detect curvilinear shapes faster than other shapes, especially when labeled as “snakes” [19]. A recent event-related potentials (ERP) study has confirmed that snakes receive prioritized processing (i.e., larger early posterior negativity amplitudes) compared to other curvilinear animals, such as spiders, worms, and beetles [20]. Thus, it seems that curvilinear shapes quickly capture attention, especially when associated with threat, suggesting that there is an interaction between exogenous factors (i.e., specific shapes) and endogenous factors (i.e., pre-existing fear and perceived threat).

Along similar lines, a recent study investigated the impact of emotionally-arousing negative stimuli in exogenously capturing attentional resources during visual search on natural scenes (i.e., during a top-down, endogenous task). Behavioral and eye movement data suggested that emotional distractors were attended to later and for shorter durations, compared to emotional targets, suggesting effective top-down control. Moreover, functional magnetic resonance imaging (fMRI) data suggested a selective increase of insular/prefrontal connectivity to face emotional distraction [21]. Thus, this study demonstrates an interaction between attention and task-relevance using a highly ecological paradigm and various measurement methods (behavioral, eye movements, and fMRI). Different lines of research further showed that the effect of negative/threatening stimuli not only biases attention, but also has important consequences on post-perceptual/attentional processes, e.g., on short-term memory [22] and prospective memory [23]. Note that these latter “memory” effects are based on the interplay between exogenous capture of negative stimuli and endogenous task-based demand. Together, these studies suggest an interaction between endogenous and exogenous factors, as well as an interaction between attention and memory. Hence, while factors that affect attention interact, attention itself also interacts with other processes, such as expectancies, memory (sometimes in the form of a-posteriori expectancies), etc.

## 3. The Effect of Pre-Stimulus Factors on Attention Bias: Interaction between Expectancies, Uncertainty and Valence

One of the factors that affects attention is task relevance, which refers to the relevance of certain stimuli to a specific task. Some stimuli could be irrelevant and thus act as distractors, while still capturing attention and hindering performance (e.g., [24] on the effects of spider distractors on the performance of participants with spider phobia). Task relevance has also been found to play a role in specific types of attention bias: in a sample of healthy participants, Gronau and Izoutcheev [25] found that generally, task-irrelevant distractors can be successfully ignored. However, the same distractors can affect behavioral performance if they contain images from a to-be-detected category. Dodd, Vogt, Turkileri, and Notebaert [26] found that trait anxiety is associated with decreased performance on a visual search array when angry faces were used as task-irrelevant stimuli, but not when they were task-relevant. Thus, task relevance interacts with threat value and pre-existing pathology (see also [27] for similar findings among participants with anxiety).

Recent studies suggest that there are additive effects between task-relevance and a-priori expectancies. Specifically, participants were informed regarding the likelihood of encountering a certain target color out of two possible target colors amidst 4 colors (i.e., 3 distractors) in a visual search array [28]. Participants were asked to indicate the orientation of rectangular bars in target colors. The search array was preceded by a spatially uninformative cue which indicated the upcoming target color half of the time. Behavioral and ERP results (N2pc) showed that cues for both target colors attracted attention, even though participants were informed that one color was more likely to appear (80%) compared to the other color (20%). Thus, cues that act as preparatory attentional templates only reflect relevance, rather than statistical expectations regarding the upcoming targets. Additionally, results showed that during the search, expectancies regarding probabilities of target colors did have an effect, as reaction time and the onset of the N2pc component were delayed when the infrequent target color appeared [28]. Thus, relevance and a-priori encounter expectancies differentially affect attentional capture.

Along the same lines, embedding threatening stimuli in previously learned visual contexts may facilitate their detection, as opposed to threatening stimuli in new contexts and to non-threatening stimuli in old contexts [29]. The facilitative influence of visual contexts might be mediated by changes in critical expectancies. Hence, the authors concluded that participants use contextual cues in order to detect threat. In fact, there is evidence that visual contexts can shape expectancy regarding which objects are likely to appear within a given context. Thus, in a given visual context, one would expect certain objects to appear in specific places. Accordingly, one study found that objects that were placed in correct locations (e.g., an umbrella over a hoodie) were detected faster, compared to objects that were placed in incorrect locations, (e.g., a hoodie over an umbrella; [30], Experiment 1). Specifically, in Experiment 1, both objects were attended and spatial consistency (i.e., placings that made sense) led to facilitation. In a second experiment, only one of the two objects was attended (i.e., cued) and task-relevant, while the other object was unattended (i.e., uncued) and became irrelevant due to different instructions. An interaction between spatial consistency and task-relevance was found, such that the effect of spatial consistency disappeared when an object became task-irrelevant ([30]; Exp. 2). Thus, when one object leaves the focus of attention, expectancy to find a certain object in a given visual context no longer facilitates its detection. Taken together, the aforementioned studies suggest that there is an interaction between higher cognitive factors, such as task-relevance, or lower level factors, such as previously learned visual contexts, with expectancies and attention. The study of such factors and their interaction with expectancy could have important theoretical and clinical implications in better understanding attention bias and ABM procedures.

Sussman, Jin, and Mohanty [31] emphasize the role of prestimulus factors, (e.g., goals and expectations), as well as the role of the interaction between exogenous and endogenous factors, in threat-related perception and attention in anxiety (see also [32]). For instance, a-priori anticipation to encounter aversive stimuli (i.e., expectancy bias) lies at the heart of all anxiety disorders, and this bias can also interact with attention bias (for a review, see [33]). It has been suggested that inducing a-priori expectancy to encounter a certain stimulus increases the detectability of the stimulus by increasing perceptual sensitivity to its features [34]. Thus, there is an interaction between perceptual sensitivity, expectancy, and attention. Along these lines, Sussman, Szekely, Hajcak, and Mohanty (2016; [35]) emphasize the role of anticipation as it subsequently shapes perception of threat, which is enhanced in anxiety. Such perceptions of threat may also affect attention allocation toward threat. Several studies have already integrated expectancy cues that predict the threat value of the subsequent stimulus into attention bias paradigms, thus advocating for more integrative research (dot probe: [36]; visual search: [37,38,39,40]).

Aue et al. [38] manipulated expectancy in order to measure its effects on attention bias in spider phobia. Specifically, participants were presented with a visual search array, which included 9 pictures: 8 neutral distractors (butterflies) and 1 deviant target, which was either neutral (i.e., birds) or threatening (i.e., spiders). Participants were asked to detect the deviant picture as quickly and as accurately as possible. Prior to the presentation of the search array, a verbal cue appeared, indicating the likelihood of the appearance of a specific target (“bird 90%”, “spider 90%”, or “bird spider 50%”). In fact, the first two types of cues were valid on 71% of trials. Results showed that a-priori expectancies (i.e., cues) strongly affected the detection of neutral stimuli (i.e., faster reactions on congruent trials compared to incongruent trials in which bird targets appeared). Notably though, it did not affect detection of spider targets in either group of participants (with and without spider phobia), when both types of targets appeared equally often. Thus, a-priori expectancies differentially affected the detection of each type of target (birds vs. spiders). Further correlational findings in another study, nonetheless, suggest that there is an association between expectancy bias and attention bias for threatening stimuli ([41]; for a review, see [10]), but causality in this association needs further investigation.

Follow-up studies suggest that in fear of spiders, there is a competition between valence and certainty. Specifically, expectancy was manipulated in the same way: using a verbal cue indicating the probability of encountering one of two targets in a following visual search array, with either bird or spider targets. Here, however, the frequency of the appearance of each target was manipulated, while keeping the cue-target congruency rate constant (i.e., 71%). When bird targets appeared more often than spider targets in the same paradigm, both low and high fear groups exhibit reduced attention bias toward spiders and reacted according to the cues ([37]; Experiment 1 and 2). This conclusion is also in line with the finding that ABM-positive-search training, in which participants are trained to attend to positive stimuli, could be more effective than negative-search-training, in which participants are trained to attend to negative stimuli. This could be due to the fact that positive-search training encourages participants to actively use goal-directed inhibitory control, thus ignoring task-irrelevant threatening stimuli and focusing on more positive, task-relevant targets [42,43]. Additionally, when spiders appeared more often than birds, a reduction of attention bias was also found, but not as strongly as in Experiments 1 and 2 in which bird targets appeared more often ([37]; Experiment 3). As reduction of attention bias was found when either target (bird or spider) appeared more often, and both were task-relevant each time, it is possible that frequency of a specific target indicates certainty: when both targets appear equally often, participants feel the least certain about encountering any specific stimulus and consequently do not use the cues. However, when either target appears more often, participants may feel more certain that they will encounter a specific target and thus exhibit a reaction that is more in line with the cues.

Using the affective cueing paradigm, an ERP study, Gole, Schäfer, and Schienle [44], manipulated certainty using three types of conditions: certainty-negative (i.e., the negative cue is always followed by a negative picture, certainty-neutral (i.e., the neutral cue is always followed by a neutral picture), and uncertainty (i.e., uncertain cue followed by either a negative or a neutral picture). In addition, two groups of participants were included: low and high in intolerance of uncertainty (IU). This measure reflects participants’ ability to cope with life’s various uncertainties about the future [45]. Results showed that during exposure in the later time windows (late positive potentials; LPP2: 750–1000 ms), an interaction emerged between cue (certain, uncertain), category of outcome (aversive, neutral) and group (low IU, high IU), such that only among high-IU participants, processing of aversive pictures was affected by the type of the preceding cue. Concretely, in high IU participants, LPP2 amplitudes were smaller following the uncertain cue-aversive picture condition, compared to the certainly negative condition. The authors concluded that uncertain cues received heightened allocation of resources, thus making these resources less available for emotional processing during exposure [44]. In this study, uncertainty was measured as a trait quality (IU) and predictability was manipulated as an experimental factor (see also Reference [46]).

Using a similar paradigm and shorter anticipation phases, Lin et al. [47] found that valence (positive and negative pictures) modulates the effect of IU on allocation of attentional resources when viewing emotional pictures, such that during early sensory processes (P2: 130–180 ms), experimental uncertainty reduces attention to negative pictures, and enhances it during later sensory processes (N2: 220–300 ms). Experimental uncertainty did not affect attention toward positive pictures. Thus, uncertainty only affected attention toward negative stimuli. While Gole et al. [44] found increased attentional resources during uncertain anticipation, Lin et al. [47] found that the valence of subsequent stimuli further modulates this effect. Hence, certainty/expectancy and valence/threat value seem to interact during emotional processing and allocation of attentional resources

As threat can affect attention bias, so can reward. For instance, Kress et al. [48] found that optimistic expectancies can affect attention. Specifically, verbal cues indicated the likelihood of encountering certain stimuli, which were associated with either monetary loss or gain (i.e., reward). Results showed that the effect of optimistic expectancies on attention deployment was stronger than the effect of pessimistic expectancies. Importantly, this finding was found among a sample of healthy participants, and so these findings might differ when presented to anxious participants, especially if the presented stimuli are disorder-relevant (e.g., spiders to participants with spider phobia).

In the domain of expectancy and attention bias toward positive stimuli, there is evidence for bidirectional causality. It has recently been established that a-priori optimistic expectancies affect positive attention bias [48], and that ABM toward positive stimuli can enhance optimistic expectancies ([49]; see [50] for suggested neurobiological mechanisms underlying such interplay between the biases). Considering many shared factors between negative and positive emotions, the bidirectional causality in the context of positive stimuli suggests that it might also exist in the case of negative stimuli (for reviews on expectancy and attention bias toward positive stimuli, see [13,51]).

Reward can further interact with other factors, such as prior attentional history, which is the history of attentional allocation toward certain items, and has also been found to play a role in visual attention (for a review, see [52]). For instance, if participants had been trained to shift their gaze away from a certain feature due to motivational reasons, (i.e., monetary reward), they would find it difficult to direct their attention away from the same feature once again on subsequent tasks, even if this feature is now task-irrelevant and this behavior conflicts with the new tasks’ goals ([53]; Experiment 1). In other words, although participants were trained to look away from certain features, they found it difficult to do it again on a different task, as they have learned that the same specific feature is related to a reward. Thus, prior history and prior motivation can interact and bias current orienting and direction of visual attention. Hence, factors that affect the interaction between expectancies an attention may also interact with other, seemingly unrelated factors, creating a complex network of interactions.

To summarize, many variables could affect the interaction between expectancy and attention bias toward threat. Specifically, while some factors (e.g., task-relevance, salience,) are more directly related to expectancy, other factors (e.g., attentional history) have not been directly examined in this context. A thorough manipulation of these factors can lead to a more comprehensive understanding of expectancy, attention bias, and their interaction in health and in anxiety disorders. Importantly, these factors cannot be easily divided into top-down or bottom-up, as attentional history can be affected by both processes. Similarly, salience could be considered a bottom-up factor, but it also interacts with pre-existing pathologies and other individual differences.

## 4. The Role of Individual Differences in the Expectancy-Attention Interaction

Pre-existing pathology can also affect expectancies and attention bias. For instance, whereas control participants showed an attention bias toward positive distractors and no bias toward negative distractors, anxious participants exhibited attention bias toward negative distractors although these were task-irrelevant, and not towards positive distractors [27]. Depressed participants did not exhibit an attention bias toward either negative or positive distractors [27]. Similarly, sensitivity to and response criteria for emotional faces were suggested as independent factors which influence perceptual sensitivity in individuals with social anxiety [54]. This sensitivity was specific for mildly angry faces and was not found toward mildly happy faces. These findings also imply an interpretation bias, in that anxious participants interpret neutral stimuli as threatening. A recent study has also simultaneously assessed the roles of attention, memory, and evaluation processes in health anxiety [55]. It was found that compared to participants with depression and to healthy participants, participants with pre-existing pathological health anxiety exhibited stronger attention bias to symptom- and illness-related words, more biased recognition of health-threat-related words, and more negative explicit evaluations of health threat.

Moreover, attention bias varies within anxiety disorders and even within specific phobias. For instance, participants with spider phobia and participants with blood-injection-injury (BII) phobia differ in terms of attention bias patterns. Oftentimes, general hypervigilance is found in BII phobia (e.g., [56]), while at other times, avoidance is found (e.g., [57]). Similarly, BII phobia differs from spider phobia in terms of expectancies and in terms of disgust and disgust sensitivity, as disgust is suggested to be the predominant emotion in BII phobia, while fear is suggested to be the more dominant emotion in other specific phobias (for a review, see [58]). Lastly, it has been suggested that attention bias in specific phobias may differ from attention bias in generalized anxiety [59].

A-priori and a-posteriori expectancies seem to differ between small animal phobias and BII phobia. For instance, Pury and Mineka [60] randomly paired aversive or neutral outcomes (shock, neutral tone, no outcome) with BII-related and neutral pictures. These were presented to participants with low and high BII fear level. Results showed that post-experimentally, all participants (i.e., with low and high BII fear levels) equally overestimated the co-occurrence of BII-related pictures with a shock, indicating the existence of a-posteriori/memory bias. De Jong and Peters [61] found somewhat different results. They presented neutral and BII-related pictures with different outcomes: no consequences (neutral), drinking 5 mL of harmless but bad tasting solution (disgust), or receiving an electrical shock (harm-related). Participants were low and high in BII fear. A-priori consequence bias was found, such that all participants overestimated the likelihood of co-occurrence between BII-related pictures and disgust- and harm-related outcomes. (See [62] for similar results using a thought experiment. Of note, in this study, the bias was stronger in the high BII fear group, but still present in the low fear group.) However, all participants went through online adjustment. In other words, they managed to change their expectation during the experiment, between a-priori and a-posteriori measurements, as a-posteriori expectancy bias was not found. Thus, participants underwent a learning process in which they (likely unconsciously) realized that stimuli were randomly paired with outcomes.

As mentioned earlier, disgust and disgust sensitivity have been found to affect fear reactions in specific phobias, as well as in non-clinical individuals. For instance, among a non-clinical sample, it was found that spiders, snakes and parasites induced strong feelings of disgust and fear [63]. Across participants, a strong correlation emerged between reported fear and disgust ratings of the animals. In addition, less fearful participants rated (harmless) grass snakes as less fear-evoking than vipers, compared to snake fearful participants. Thus, the authors conclude that this effect of overgeneralization of fear to harmless animals can be used as a clinical screening tool for animal phobia [63].

Disgust may be considered a unique emotion. Specifically, using the dot-probe paradigm, a recent ERP study found that unlike fear and anger, disgust leads to avoidance for evolutionary reasons, as it diverts attention away from the relevant stimulus, rather than maintains it [64]. This difference was greater among participants with high disgust sensitivity. Thus, disgust and disgust sensitivity affect emotional reactions toward threatening stimuli, which also depend on preexisting anxiety, perhaps due to evolutionary reasons. The authors suggest a model, according to which early sensory processes enhance the perception of fear and anger while suppressing disgust, while later processes regulate emotion and behavior.

To summarize, both attention and expectancy biases differ in different anxiety disorders, even within specific phobias. However, the interaction between both biases has yet to be studied extensively in different disorders. Thus, it is important to take existing psychopathologies into account when studying expectancies and attention and when attempting to treat attention bias more effectively.

## 5. Conclusions and Future Directions

The current review presents recent studies that have examined factors which affect expectancies and attention bias. Specifically, these studies suggest that a-priori expectancies can have various effects on attention bias toward threatening stimuli in anxiety. Importantly, expectancies can be manipulated in various ways, and thus different factors can affect the interaction between expectancies and attention, such as IU, pre-existing anxiety, task-relevance, and visual context. We present these findings as examples of possible factors, out of many, which could affect attention bias in a dynamic network, with a focus on a-priori expectancies. As Parsons et al. [7] demonstrated, the interaction between cognitive biases can be examined more systematically, using a moderated network modelling approach. The study of the interaction between various factors that affect attention bias could also benefit from a similar line of study, especially if causality can also be included. Consequently, the study of interactions between biases and specifically between expectancy and attention bias can also have clinical implications.

As attention in general and attention bias in anxiety in particular are vastly studied, an integratory line of research may be of great importance. Cognitive biases do not exist in a vacuum, and no singular characteristic or bias can help in characterizing different disorders. For instance, cognitive biases are differentially expressed in anxiety and in depression [12]. Specifically, Richter et al. [12] developed a battery that simultaneously examines different cognitive biases in anxiety and in depression. Thus, both disorders were differentially characterized in terms of the various cognitive biases involved. This differentiation can assist to develop better diagnostic treatments and tools for anxiety, depression, and other psychiatric disorders. In line with the current review, Richter et al. [12] suggested that such a battery can be a support system for diagnoses and treatment possibilities and that developing such protocols for treatment can help in the diagnostic process. Such a line of research also complements other recent studies which focused on the integration of various cognitive biases in cognitive disorders (e.g., [65,66,67]). Future studies can develop similar batteries for various disorders, with an emphasis on expectancies, especially in anxiety disorders [33].

As research integrates more elements, a more holistic view can be formed regarding these patterns of biased processing of negative emotional stimuli. Similarly, as research considers additional factors that influence the processing of emotional stimuli, a fuller picture can be formed. This understanding of the deeper complexities of emotional processing can help in developing more fine-tuned treatments and cognitive training options. Such options include machine learning, which tailors training according to each individual’s needs [68]. Another option involves ABM, as it aims at reducing anxiety by reducing attention bias and vice versa (for a review, see [3]). Specifically, ABM trains participants to attend to specific stimuli, thereby reducing attention bias to threatening and anxiety-related stimuli. This process usually involves a manipulation of frequency of the appearance of threatening and neutral stimuli in order to increase reactions aimed to attend or avoid threat (for review on ABM, [43]). We propose that one way of achieving that is by inducing expectancies and certainty (e.g., frequency, predictability) during training, as both factors can be “goal-directed” and “salience-driven”. We suggest that the interaction of attention bias with other cognitive, motivational, affective, and personality factors and biases (e.g., expectancy bias) may help explain greater variance in emotional responses and may be of use when developing cognitive training.

A recent review has suggested that ABM can be very unreliable (e.g., [43]). As McNally [69] has pointed out, this crisis of unreliability can be an opportunity for growth. The world of ABM and cognitive training is expanding into new and innovative directions, which include the integration of perception, learning, and memory with individual differences [70] and individually tailored trainings based on machine learning [68].

To summarize, we have briefly reviewed recent literature showing that there is a mutual influence between different cognitive biases and between different cognitive, motivational, affective, and personality factors. Findings suggest that many of these interactions play a role in emotional responses and in attention bias. We propose that effective training of attention can be established using a manipulation of other factors, such as expectancy, which could be added to manipulation of attention bias in ABM. Further research and advanced analysis strategies could establish innovative trainings, which in turn may also shed light on the complexities of emotional responses and attention bias toward anxiety-relevant stimuli.

## References

[1] Abado E., Richter T., Okon-Singer H. (2020). Attention bias toward negative stimuli. Cognitive Biases in Health and Psychiatric Disorders.

[2] Okon-Singer H. (2018). The role of attention bias to threat in anxiety: Mechanisms, modulators and open questions. Curr. Opin. Behav. Sci..

[3] Van Bockstaele B., Verschuere B., Tibboel H., De Houwer J., Crombez G., Koster E.H.W. (2014). A review of current evidence for the causal impact of attentional bias on fear and anxiety. Psychol. Bull..

[4] Cisler J.M., Koster E.H.W. (2010). Mechanisms of attentional biases towards threat in anxiety disorders: An integrative review. Clin. Psychol. Rev..

[5] Klein A.M., van Niekerk R., ten Brink G., Rapee R.M., Hudson J.L., Bögels S.M., Becker E.S., Rinck M. (2017). Biases in attention, interpretation, memory, and associations in children with varying levels of spider fear: Inter-relations and prediction of behavior. J. Behav. Ther. Exp. Psychiatry.

[6] Nieuwenhuys A., Oudejans R.R. (2017). Anxiety and performance: Perceptual-motor behavior in high-pressure contexts. Curr. Opin. Psychol..

[7] Parsons S., Songco A., Booth C., Fox E. (2019). Emotional Information-Processing Correlates of Positive Mental Health in Adolescence: A Network Analysis Approach. https://psyarxiv.com/8ygnh/.

[8] Everaert J., Duyck W., Koster E.H.W. (2014). Attention, interpretation, and memory biases in subclinical depression: A proof-of-principle test of the combined cognitive biases hypothesis. Emotion.

[9] Hirsch C.R., Clark D.M., Mathews A. (2006). Imagery and Interpretations in Social Phobia: Support for the Combined Cognitive Biases Hypothesis. Behav. Ther..

[10] Aue T., Okon-Singer H. (2015). Expectancy biases in fear and anxiety and their link to biases in attention. Clin. Psychol. Rev..

[11] Marchetti I., Everaert J., Dainer-Best J., Loeys T., Beevers C.G., Koster E.H.W. (2018). Specificity and overlap of attention and memory biases in depression. J. Affect. Disord..

[12] Richter T., Fishbain B., Markus A., Richter-Levin G., Okon-Singer H. (2020). Using machine learning-based analysis for behavioral differentiation between anxiety and depression. Sci. Rep..

[13] Dricu M., Kress L., Aue T. (2020). The neurophysiological basis of optimism bias. Cognitive Biases in Health and Psychiatric Disorders.

[14] de Jong P.J., Daniels J.K. (2020). Negative expectancy biases in psychopathology. Cognitive Biases in Health and Psychiatric Disorders.

[15] Okon-Singer H., Lichtenstein-Vidne L., Cohen N. (2013). Dynamic modulation of emotional processing. Biol. Psychol..

[16] Mogg K., Bradley B.P. (2018). Anxiety and Threat-Related Attention: Cognitive-Motivational Framework and Treatment. Trends Cogn. Sci..

[17] Awh E., Belopolsky A.V., Theeuwes J. (2012). Top-down versus bottom-up attentional control: A failed theoretical dichotomy. Trends Cogn. Sci..

[18] Öhman A., Flykt A., Esteves F. (2001). Emotion drives attention: Detecting the snake in the grass. J. Exp. Psychol. Gen..

[19] LoBue V. (2014). Deconstructing the snake: The relative roles of perception, cognition, and emotion on threat detection. Emotion.

[20] Van Strien J.W., Christiaans G., Franken I.H.A., Huijding J. (2016). Curvilinear shapes and the snake detection hypothesis: An ERP study: Curvilinear shapes and snake detection. Psychophysiol.

[21] Pedale T., Macaluso E., Santangelo V. (2019). Enhanced insular/prefrontal connectivity when resisting from emotional distraction during visual search. Brain Struct. Funct..

[22] Buttafuoco A., Pedale T., Buchanan T.W., Santangelo V. (2018). Only “efficient” emotional stimuli affect the content of working memory during free-recollection from natural scenes. Cogn. Process..

[23] Pedale T., Basso D., Santangelo V. (2017). Processing of negative stimuli facilitates event-based prospective memory only under low memory load. J. Cogn. Psychol..

[24] Okon-Singer H., Alyagon U., Kofman O., Tzelgov J., Henik A. (2011). Fear-related pictures deteriorate the performance of university students with high fear of snakes or spiders. Stress.

[25] Gronau N., Izoutcheev A. (2017). The necessity of visual attention to scene categorization: Dissociating “task-relevant” and “task-irrelevant” scene distractors. J. Exp. Psychol. Hum. Percept. Perform..

[26] Dodd H.F., Vogt J., Turkileri N., Notebaert L. (2017). Task relevance of emotional information affects anxiety-linked attention bias in visual search. Biol. Psychol..

[27] Lichtenstein-Vidne L., Okon-Singer H., Cohen N., Todder D., Aue T., Nemets B., Henik A. (2017). Attentional bias in clinical depression and anxiety: The impact of emotional and non-emotional distracting information. Biol. Psychol..

[28] Berggren N., Eimer M. (2019). The roles of relevance and expectation for the control of attention in visual search. J. Exp. Psychol. Hum. Percept. Perform..

[29] Szekely A., Rajaram S., Mohanty A. (2017). Context learning for threat detection. Cogn. Emot..

[30] Gronau N., Shachar M. (2014). Contextual integration of visual objects necessitates attention. Atten. Percept. Psychophys..

[31] Sussman T.J., Jin J., Mohanty A. (2016). Top-down and bottom-up factors in threat-related perception and attention in anxiety. Biol. Psychol..

[32] Sussman T.J., Jin J., Mohanty A. (2020). The impact of top-down factors on threat perception biases in health and anxiety. Cognitive Biases in Health and Psychiatric Disorders.

[33] Grupe D.W., Nitschke J.B. (2013). Uncertainty and anticipation in anxiety: An integrated neurobiological and psychological perspective. Nat. Rev. Neurosci..

[34] Stein T., Peelen M.V. (2015). Content-specific expectations enhance stimulus detectability by increasing perceptual sensitivity. J. Exp. Psychol. Gen..

[35] Sussman T.J., Szekely A., Hajcak G., Mohanty A. (2016). It’s all in the anticipation: How perception of threat is enhanced in anxiety. Emotion.

[36] Gladwin T.E., Möbius M., McLoughlin S., Tyndall I. (2019). Anticipatory versus reactive spatial attentional bias to threat. Br. J. Psychol..

[37] Abado E., Sagi J., Silber N., De Houwer J., Aue T., Okon-Singer H. (2020). Reducing attention bias in spider fear by manipulating expectancies. Behav. Res. Ther..

[38] Aue T., Guex R., Chauvigné L.A.S., Okon-Singer H. (2013). Varying expectancies and attention bias in phobic and non-phobic individuals. Front. Hum. Neurosci..

[39] Aue T., Chauvigné L.A.S., Bristle M., Okon-Singer H., Guex R. (2016). Expectancy influences on attention to threat are only weak and transient: Behavioral and physiological evidence. Biol. Psychol..

[40] Aue T., Guex R., Chauvigné L.A.S., Okon-Singer H., Vuilleumier P. (2019). Expectancies influence attention to neutral but not necessarily to threatening stimuli: An fMRI study. Emotion.

[41] Aue T., Hoeppli M.-E., Piguet C., Sterpenich V., Vuilleumier P. (2013). Visual avoidance in phobia: Particularities in neural activity, autonomic responding, and cognitive risk evaluations. Front. Hum. Neurosci..

[42] Mogg K., Bradley B.P. (2016). Anxiety and attention to threat: Cognitive mechanisms and treatment with attention bias modification. Behav. Res. Ther..

[43] Mogg K., Waters A.M., Bradley B.P. (2017). Attention Bias Modification (ABM): Review of Effects of Multisession ABM Training on Anxiety and Threat-Related Attention in High-Anxious Individuals. Clin. Psychol. Sci..

[44] Gole M., Schäfer A., Schienle A. (2012). Event-related potentials during exposure to aversion and its anticipation: The moderating effect of intolerance of uncertainty. Neurosci. Lett..

[45] Carleton R.N., Norton M.A.P.J., Asmundson G.J.G. (2007). Fearing the unknown: A short version of the Intolerance of Uncertainty Scale. J. Anxiety Disord..

[46] Morriss J., McSorley E., van Reekum C.M. (2018). I don’t know where to look: The impact of intolerance of uncertainty on saccades towards non-predictive emotional face distractors. Cogn. Emot..

[47] Lin H., Jin H., Liang J., Yin R., Liu T., Wang Y. (2015). Effects of Uncertainty on ERPs to Emotional Pictures Depend on Emotional Valence. Front. Psychol..

[48] Kress L., Bristle M., Aue T. (2018). Seeing through rose-colored glasses: How optimistic expectancies guide visual attention. PLoS ONE.

[49] Kress L., Aue T. (2019). Learning to Look at the Bright Side of Life: Attention Bias Modification Training Enhances Optimism Bias. Front. Hum. Neurosci..

[50] Kress L., Aue T. (2017). The link between optimism bias and attention bias: A neurocognitive perspective. Neurosci. Biobehav. Rev..

[51] Vogt J., Bajandouh Y., Alzubaidi U. (2020). Beyond negativity: Motivational relevance as cause of attentional bias to positive stimuli. Cognitive Biases in Health and Psychiatric Disorders.

[52] Wolfe J.M. (2019). Visual Attention: The Multiple Ways in which History Shapes Selection. Curr. Biol..

[53] Anderson B.A., Kim H. (2019). On the relationship between value-driven and stimulus-driven attentional capture. Atten. Percept. Psychophys..

[54] Yoon K.L., Yang J.-W., Chong S.C., Oh K.J. (2014). Perceptual Sensitivity and Response Bias in Social Anxiety: An Application of Signal Detection Theory. Cogn. Ther. Res..

[55] Witthöft M., Kerstner T., Ofer J., Mier D., Rist F., Diener C., Bailer J. (2016). Cognitive Biases in Pathological Health Anxiety: The Contribution of Attention, Memory, and Evaluation Processes. Clin. Psychol. Sci..

[56] Buodo G., Peyk P., Junghöfer M., Palomba D., Rockstroh B. (2007). Electromagnetic indication of hypervigilant responses to emotional stimuli in blood-injection-injury fear. Neurosci. Lett..

[57] Armstrong T., Hemminger A., Olatunji B.O. (2013). Attentional bias in injection phobia: Overt components, time course, and relation to behavior. Behav. Res. Ther..

[58] Çavuşoğlu M., Dirik G. (2011). Fear or disgust? The role of emotions in spider phobia and blood-injection-injury phobia. Turk Psikiyatr. Derg. Turk. J. Psychiatry.

[59] Mogg K., Bradley B., Miles F., Dixon R. (2004). BRIEF REPORT Time course of attentional bias for threat scenes: Testing the vigilance-avoidance hypothesis. Cogn. Emot..

[60] Pury C.L.S., Mineka S. (1997). Covariation bias for blood-injury stimuli and aversive outcomes. Behav. Res. Ther..

[61] de Jong P.J., Peters M.L. (2007). Blood-injection-injury fears: Harm- vs. disgust-relevant selective outcome associations. J. Behav. Ther. Exp. Psychiatry.

[62] van Overveld W.J.M., de Jong P.J., Peters M.L. (2009). Digestive and cardiovascular responses to core and animal-reminder disgust. Biol. Psychol..

[63] Polák J., Rádlová S., Janovcová M., Flegr J., Landová E., Frynta D. (2020). Scary and nasty beasts: Self-reported fear and disgust of common phobic animals. Br. J. Psychol..

[64] Zhang D., Liu Y., Wang L., Ai H., Luo Y. (2017). Mechanisms for attentional modulation by threatening emotions of fear, anger, and disgust. Cogn. Affect. Behav. Neurosci..

[65] Derakhshan N. (2020). Attentional control and cognitive biases as determinants of vulnerability and resilience in anxiety and depression. Cognitive Biases in Health and Psychiatric Disorders.

[66] Everaert J., Koster E.H.W. (2020). The interplay among attention, interpretation, and memory biases in depression: Revisiting the combined cognitive bias hypothesis. Cognitive Biases in Health and Psychiatric Disorders.

[67] Ginat-Frolich R., Shechner T. (2020). Cognitive biases across development: A detailed examination of research in fear learning. Cognitive Biases in Health and Psychiatric Disorders.

[68] Shani R., Tal S., Zilcha-Mano S., Okon-Singer H. (2019). Can Machine Learning Approaches Lead Toward Personalized Cognitive Training?. Front. Behav. Neurosci..

[69] McNally R.J. (2019). Attentional bias for threat: Crisis or opportunity?. Clin. Psychol. Rev..

[70] Dolcos F., Katsumi Y., Moore M., Berggren N., de Gelder B., Derakshan N., Hamm A.O., Koster E.H.W., Ladouceur C.D., Okon-Singer H. (2020). Neural correlates of emotion-attention interactions: From perception, learning, and memory to social cognition, individual differences, and training interventions. Neurosci. Biobehav. Rev..

